# Ethyl (2*E*,4*E*)-5-(3-bromo­phenyl­sulfon­yl)penta-2,4-dienoate

**DOI:** 10.1107/S1600536813005771

**Published:** 2013-03-06

**Authors:** V. Sabari, Ulaganathan Sankar, Ramakrishnan Uma, S. Aravindhan

**Affiliations:** a Department of Physics, Presidency College, Chennai 600 005, India; b Department of Chemistry, Pachaiyappa’s College, Chennai 600 030, India

## Abstract

In the title compound, C_13_H_13_BrO_4_S, both C=C double bonds adopt an *E* conformation. The S atom has a distorted tetra­hedral geometry with bond angles ranging from 102.17 (13) to 119.77 (14)°. The ethyl acrylate substituent adopts an extented conformation with all torsion angles close to 180°. In the crystal, mol­ecules are linked into centrosymmetric *R*_2_^2^(14) dimers *via* pairs of C—H⋯O hydrogen bonds.

## Related literature

For the biological activity of phenyl sulfonyl-containing compounds, see: De-Benedetti *et al.* (1985 ▶); Chumakov *et al.* (2006 ▶); Kremer *et al.* (2006 ▶). For related structures, see: Li *et al.* (2011 ▶); Sankar *et al.* (2012 ▶); Chakkaravarthi *et al.* (2008 ▶); Rodriguez *et al.* (1995 ▶). For graph-set analysis of hydrogen bonds, see: Sankar *et al.* (2012 ▶).
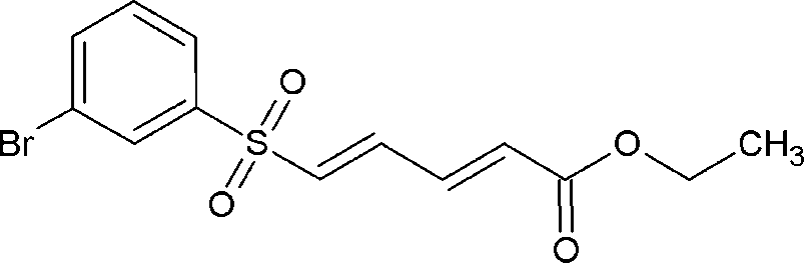


## Experimental

### 

#### Crystal data

C_13_H_13_BrO_4_S
*M* *_r_* = 345.20
Monoclinic, 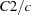

*a* = 27.883 (5) Å
*b* = 6.001 (5) Å
*c* = 17.256 (5) Å
β = 94.020 (5)°
*V* = 2880 (3) Å^3^
*Z* = 8
Mo *K*α radiation
μ = 3.01 mm^−1^
*T* = 293 K
0.32 × 0.20 × 0.10 mm

#### Data collection

Bruker APEXII CCD area-detector diffractometer
Absorption correction: multi-scan (*SADABS*; Bruker, 2008 ▶) *T*_min_ = 0.972, *T*_max_ = 0.992
14906 measured reflections
3479 independent reflections
2360 reflections with *I* > 2σ(*I*)
*R*_int_ = 0.035

#### Refinement

*R*[*F* ^2^ > 2σ(*F* ^2^)] = 0.039
*wR*(*F* ^2^) = 0.116
*S* = 1.01
3479 reflections
172 parameters
H-atom parameters constrained
Δρ_max_ = 0.73 e Å^−3^
Δρ_min_ = −0.41 e Å^−3^

### 

Data collection: *APEX2* (Bruker, 2008 ▶); cell refinement: *SAINT* (Bruker, 2008 ▶); data reduction: *SAINT*; program(s) used to solve structure: *SHELXS97* (Sheldrick, 2008 ▶); program(s) used to refine structure: *SHELXL97* (Sheldrick, 2008 ▶); molecular graphics: *ORTEP-3 for Windows* (Farrugia, 2012 ▶); software used to prepare material for publication: *SHELXL97* and *PLATON* (Spek, 2009 ▶).

## Supplementary Material

Crystal structure: contains datablock(s) I, global. DOI: 10.1107/S1600536813005771/bt6881sup1.cif

Structure factors: contains datablock(s) I. DOI: 10.1107/S1600536813005771/bt6881Isup2.hkl

Supplementary material file. DOI: 10.1107/S1600536813005771/bt6881Isup3.cml

Additional supplementary materials:  crystallographic information; 3D view; checkCIF report

## Figures and Tables

**Table 1:** Hydrogen-bond geometry (Å, °)

*D*—H⋯*A*	*D*—H	H⋯*A*	*D*⋯*A*	*D*—H⋯*A*
C7—H7⋯O3^i^	0.93	2.37	3.235 (4)	154

Symmetry code: (i) 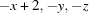.

**Fig. 1.**
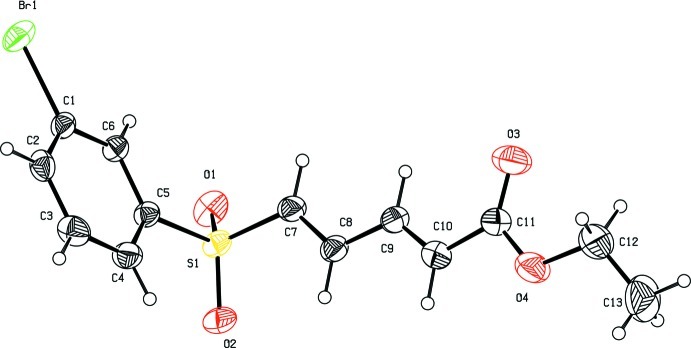
The molecular structure of the title compound, showing 30% probability displacement ellipsoids for involed H atoms.

**Fig. 2.**
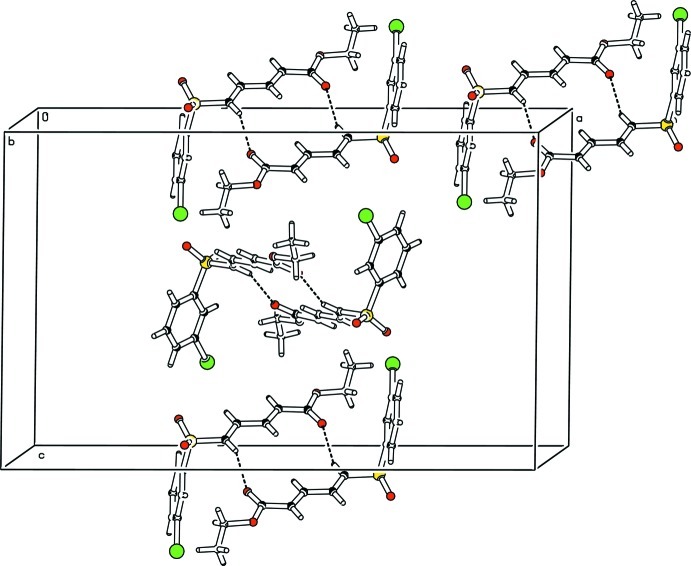
A view of the crystal packing H atoms involved in hydrogen bonding (dashed lines) have been omitted for clarity.

## supplementary crystallographic information

### Comment

Phenyl sulfonyl containing compounds show a wide range of biological properties (De-Benedetti *et al.*, 1985). Sulfonamide derivatives are extensively used in medicine as they possess a wide range of medicinal, pharmacological and antimicrobial properties (Chumakov *et al.*, 2006, Kremer *et al.*, 2006).

Fig.1. shows a displacement ellipsoid plot of the title compound. The geometric parameters of the molecule of (I) (Fig. 1) agree well with the reported values of similar structures (Sankar *et al.*, 2012). Both C=C double bonds display an E configuration. The title molecule exhibits structural similarities with the already reported related structures (Li *et al.*, 2011; Sankar, *et al.*, 2012). The dihedral angle between two planes (C6—C5—S1—O1) and (C4—C5—S1—O2) is 35.30 (13)°. The torsion angles C6—C5—S1-O1 and C4—C5—S1—O2 [-24.1 (2)° and 28 (2)°, respectively] indicate *syn*-conformation of the sulfonyl moiety. The S atom exhibits significant deviation from a regular tetrahedron, with the largest deviations being seen for the O1—S1—O2 [119.77 (14)°] and C5—S1—C7 [102.17 (13)°] angles. The widening of the angles may be due to repulsive interactions between the two short S=O bonds, similar to what is observed in related structures (Chakkaravarthi *et al.*, 2008; Rodriguez *et al.*, 1995). The ethyl acrylate group substituted at C7 position of the phenyl sulfonyl takes up an extented conformation which is evident from the torsion angle values [C8—C9—C10—C11 =] -178.6 (2)°; [C9—C10—C11—O3 =] 0.9 (3)°; [C9—C10—C11—O4 =]- 177.5 (2)°; [C10—C11—O4—C12 =]176.7 [C11—O4—C12—C13=] -156.6 (2)°.

The crystal packing is stabilized by C—H···O intermolecular interactions. The molecules are linked into centrosymmetric *R*^2^_2_(14) dimers *via* C7—H7···O3 hydrogen bonds (Table 1). The packing of the compound is shown in Fig. 2.

### Experimental

LHMDS (6.8 ml, 7.2 mmol, 2.5 equiv, 1.06 molar solution in THF) was added drop wise to a -15 °C cooled solution of bis 3-bromo phenyl sulfonyl methane (1 g, 2.9 mmol, 1 equiv) in dried THF (15 ml) under argon atm. The reaction mixture was stirred at -15 °C for 1 h, and then *trans* ethyl 4-bromo crotonate (0.61 g, 3.2 mmol, 1.1 equiv) in dry THF (5 ml) was added drop wise over the period of 10 min and allow the reaction mixture to come RT over the period of 1–2 h and stirred at RT for 24 h. The reaction mixture was quenched with sat NH_4_Cl (20 ml) and extracted with ethyl acetate (2x20 ml) washed with water (2x20 ml) and sat brine (20 ml), the organic layer was dried over MgSO_4_. Evaporation of the solvent under vacuum furnished desired crude product, The residue was purified by column chromatography on silica gel (230–400 mesh) with 17–20% of ethyl acetate in hexanes afforded the corresponding product 2E,4E)-ethyl 5-(3-bromophenylsulfonyl)penta-2,4-dienoateenoate as a colourless solid.

### Refinement

Hydrogen atoms were placed in calculated positions with C—H ranging from 0.93 Å to 0.97 Å and refined using a the riding model with fixed isotropic displacement parameters: *U*_iso_(H) = 1.5 *U*_eq_(C) for the methyl group and *U*_iso_(H) = 1.2 *U*_eq_(C) for other groups.

### Crystal data

**Table:**

C_13_H_13_BrO_4_S	*F*(000) = 1392
*M**_r_* = 345.20	*D*_x_ = 1.592 Mg m^−^^3^
Monoclinic, *C*2/*c*	Mo *K*α radiation, λ = 0.71073 Å
Hall symbol: -C 2yc	Cell parameters from 5710 reflections
*a* = 27.883 (5) Å	θ = 1.8–28.5°
*b* = 6.001 (5) Å	µ = 3.01 mm^−^^1^
*c* = 17.256 (5) Å	*T* = 293 K
β = 94.020 (5)°	Monoclinic, colourless
*V* = 2880 (3) Å^3^	0.32 × 0.20 × 0.10 mm
*Z* = 8	

### Data collection

**Table:**

Bruker APEXII CCD area-detector diffractometer	3479 independent reflections
Radiation source: fine-focus sealed tube	2360 reflections with *I* > 2σ(*I*)
Graphite monochromator	*R*_int_ = 0.035
ω and φ scans	θ_max_ = 28.1°, θ_min_ = 2.4°
Absorption correction: multi-scan (*SADABS*; Bruker, 2008)	*h* = −35→36
*T*_min_ = 0.972, *T*_max_ = 0.992	*k* = −7→7
14906 measured reflections	*l* = −22→22

### Refinement

**Table:**

Refinement on *F*^2^	Primary atom site location: structure-invariant direct methods
Least-squares matrix: full	Secondary atom site location: difference Fourier map
*R*[*F*^2^ > 2σ(*F*^2^)] = 0.039	Hydrogen site location: inferred from neighbouring sites
*wR*(*F*^2^) = 0.116	H-atom parameters constrained
*S* = 1.01	*w* = 1/[σ^2^(*F*_o_^2^) + (0.0677*P*)^2^ + 0.4368*P*] where *P* = (*F*_o_^2^ + 2*F*_c_^2^)/3
3479 reflections	(Δ/σ)_max_ = 0.006
172 parameters	Δρ_max_ = 0.73 e Å^−^^3^
0 restraints	Δρ_min_ = −0.41 e Å^−^^3^

### Special details

**Table:**

Geometry. All e.s.d.'s (except the e.s.d. in the dihedral angle between two l.s. planes) are estimated using the full covariance matrix. The cell e.s.d.'s are taken into account individually in the estimation of e.s.d.'s in distances, angles and torsion angles; correlations between e.s.d.'s in cell parameters are only used when they are defined by crystal symmetry. An approximate (isotropic) treatment of cell e.s.d.'s is used for estimating e.s.d.'s involving l.s. planes.
Refinement. Refinement of *F*^2^ against ALL reflections. The weighted *R*-factor *wR* and goodness of fit *S* are based on *F*^2^, conventional *R*-factors *R* are based on *F*, with *F* set to zero for negative *F*^2^. The threshold expression of *F*^2^ > σ(*F*^2^) is used only for calculating *R*-factors(gt) *etc*. and is not relevant to the choice of reflections for refinement. *R*-factors based on *F*^2^ are statistically about twice as large as those based on *F*, and *R*- factors based on ALL data will be even larger.

### Fractional atomic coordinates and isotropic or equivalent isotropic displacement parameters (Å^2^)

**Table:**

	*x*	*y*	*z*	*U*_iso_*/*U*_eq_	
Br1	0.826326 (13)	0.42424 (6)	0.247333 (16)	0.06505 (16)	
S1	0.83626 (3)	0.20959 (12)	−0.06349 (4)	0.0457 (2)	
O1	0.84030 (9)	0.4471 (3)	−0.06892 (12)	0.0624 (6)	
O2	0.80643 (9)	0.0904 (4)	−0.12028 (12)	0.0616 (6)	
O3	1.04522 (10)	−0.3791 (5)	−0.07427 (19)	0.0914 (9)	
C7	0.89422 (11)	0.0998 (5)	−0.05976 (16)	0.0494 (7)	
H7	0.9190	0.1778	−0.0331	0.059*	
O4	1.01637 (8)	−0.6642 (5)	−0.14354 (16)	0.0839 (8)	
C10	0.96335 (12)	−0.3814 (6)	−0.1170 (2)	0.0589 (8)	
H10	0.9396	−0.4653	−0.1438	0.071*	
C5	0.81767 (9)	0.1422 (4)	0.02920 (14)	0.0398 (6)	
C6	0.82704 (9)	0.2916 (4)	0.08969 (13)	0.0411 (6)	
H6	0.8414	0.4286	0.0815	0.049*	
C4	0.79667 (11)	−0.0622 (5)	0.04048 (17)	0.0520 (7)	
H4	0.7913	−0.1616	−0.0006	0.062*	
C8	0.90412 (11)	−0.0915 (5)	−0.09374 (16)	0.0484 (7)	
H8	0.8795	−0.1664	−0.1220	0.058*	
C11	1.01207 (12)	−0.4709 (6)	−0.10820 (18)	0.0582 (8)	
C2	0.79283 (10)	0.0286 (5)	0.17491 (17)	0.0507 (7)	
H2	0.7845	−0.0094	0.2245	0.061*	
C1	0.81425 (10)	0.2298 (5)	0.16221 (14)	0.0428 (6)	
C3	0.78369 (12)	−0.1169 (6)	0.11396 (18)	0.0585 (8)	
H3	0.7687	−0.2524	0.1223	0.070*	
C9	0.95162 (11)	−0.1879 (5)	−0.08865 (16)	0.0538 (8)	
H9	0.9761	−0.1053	−0.0630	0.065*	
C12	1.06419 (14)	−0.7664 (9)	−0.1426 (3)	0.0987 (14)	
H12A	1.0883	−0.6510	−0.1460	0.118*	
H12B	1.0709	−0.8463	−0.0942	0.118*	
C13	1.06654 (17)	−0.9140 (9)	−0.2053 (3)	0.1135 (18)	
H13B	1.0979	−0.9801	−0.2039	0.170*	
H13A	1.0605	−0.8342	−0.2532	0.170*	
H13C	1.0428	−1.0288	−0.2017	0.170*	

### Atomic displacement parameters (Å^2^)

**Table:**

	*U*^11^	*U*^22^	*U*^33^	*U*^12^	*U*^13^	*U*^23^
Br1	0.0838 (3)	0.0745 (3)	0.03659 (18)	0.00982 (17)	0.00276 (15)	−0.01125 (13)
S1	0.0591 (5)	0.0479 (4)	0.0299 (3)	−0.0021 (3)	0.0025 (3)	0.0021 (3)
O1	0.0920 (17)	0.0469 (13)	0.0493 (12)	0.0012 (11)	0.0120 (11)	0.0132 (9)
O2	0.0664 (14)	0.0790 (16)	0.0375 (10)	−0.0003 (11)	−0.0098 (9)	−0.0063 (9)
O3	0.0638 (17)	0.102 (2)	0.105 (2)	−0.0017 (15)	−0.0198 (15)	−0.0347 (17)
C7	0.0517 (18)	0.0586 (19)	0.0382 (14)	−0.0134 (14)	0.0044 (12)	−0.0030 (12)
O4	0.0497 (14)	0.0894 (18)	0.110 (2)	0.0094 (13)	−0.0102 (13)	−0.0372 (16)
C10	0.0491 (19)	0.064 (2)	0.0635 (19)	−0.0077 (15)	0.0030 (15)	−0.0109 (15)
C5	0.0440 (15)	0.0432 (14)	0.0321 (12)	−0.0003 (12)	0.0023 (11)	0.0022 (10)
C6	0.0452 (16)	0.0418 (15)	0.0364 (13)	−0.0002 (12)	0.0035 (11)	0.0002 (11)
C4	0.0541 (19)	0.0563 (19)	0.0453 (15)	−0.0129 (14)	0.0024 (13)	−0.0016 (13)
C8	0.0542 (18)	0.0529 (17)	0.0386 (14)	−0.0084 (14)	0.0065 (12)	−0.0040 (12)
C11	0.052 (2)	0.070 (2)	0.0517 (17)	−0.0072 (16)	0.0005 (15)	−0.0075 (15)
C2	0.0421 (17)	0.068 (2)	0.0425 (15)	0.0031 (14)	0.0089 (12)	0.0115 (13)
C1	0.0410 (15)	0.0531 (16)	0.0341 (12)	0.0095 (13)	0.0009 (11)	−0.0028 (11)
C3	0.059 (2)	0.063 (2)	0.0539 (18)	−0.0192 (16)	0.0038 (15)	0.0145 (14)
C9	0.0539 (19)	0.062 (2)	0.0456 (15)	−0.0123 (15)	0.0076 (13)	−0.0103 (14)
C12	0.053 (2)	0.134 (4)	0.107 (3)	0.027 (2)	−0.012 (2)	−0.035 (3)
C13	0.081 (3)	0.139 (5)	0.121 (4)	0.045 (3)	0.013 (3)	−0.007 (3)

### Geometric parameters (Å, º)

**Table:**

Br1—C1	1.888 (3)	C6—H6	0.9300
S1—O2	1.431 (2)	C4—C3	1.382 (4)
S1—O1	1.433 (2)	C4—H4	0.9300
S1—C7	1.742 (3)	C8—C9	1.442 (4)
S1—C5	1.763 (2)	C8—H8	0.9300
O3—C11	1.193 (4)	C2—C1	1.371 (4)
C7—C8	1.327 (4)	C2—C3	1.377 (5)
C7—H7	0.9300	C2—H2	0.9300
O4—C11	1.320 (4)	C3—H3	0.9300
O4—C12	1.467 (4)	C9—H9	0.9300
C10—C9	1.310 (5)	C12—C13	1.403 (6)
C10—C11	1.459 (5)	C12—H12A	0.9700
C10—H10	0.9300	C12—H12B	0.9700
C5—C4	1.379 (4)	C13—H13B	0.9600
C5—C6	1.387 (3)	C13—H13A	0.9600
C6—C1	1.376 (3)	C13—H13C	0.9600
			
O2—S1—O1	119.77 (14)	O3—C11—C10	124.4 (3)
O2—S1—C7	109.23 (14)	O4—C11—C10	112.9 (3)
O1—S1—C7	107.56 (15)	C1—C2—C3	119.7 (3)
O2—S1—C5	108.23 (13)	C1—C2—H2	120.2
O1—S1—C5	108.47 (13)	C3—C2—H2	120.2
C7—S1—C5	102.17 (13)	C2—C1—C6	121.8 (2)
C8—C7—S1	122.1 (2)	C2—C1—Br1	118.4 (2)
C8—C7—H7	118.9	C6—C1—Br1	119.8 (2)
S1—C7—H7	118.9	C2—C3—C4	120.3 (3)
C11—O4—C12	118.3 (3)	C2—C3—H3	119.9
C9—C10—C11	122.9 (3)	C4—C3—H3	119.9
C9—C10—H10	118.6	C10—C9—C8	125.8 (3)
C11—C10—H10	118.6	C10—C9—H9	117.1
C4—C5—C6	121.9 (2)	C8—C9—H9	117.1
C4—C5—S1	119.1 (2)	C13—C12—O4	110.3 (3)
C6—C5—S1	118.9 (2)	C13—C12—H12A	109.6
C1—C6—C5	117.6 (2)	O4—C12—H12A	109.6
C1—C6—H6	121.2	C13—C12—H12B	109.6
C5—C6—H6	121.2	O4—C12—H12B	109.6
C5—C4—C3	118.8 (3)	H12A—C12—H12B	108.1
C5—C4—H4	120.6	C12—C13—H13B	109.5
C3—C4—H4	120.6	C12—C13—H13A	109.5
C7—C8—C9	122.6 (3)	H13B—C13—H13A	109.5
C7—C8—H8	118.7	C12—C13—H13C	109.5
C9—C8—H8	118.7	H13B—C13—H13C	109.5
O3—C11—O4	122.7 (3)	H13A—C13—H13C	109.5
			
O2—S1—C7—C8	−12.3 (3)	C12—O4—C11—O3	−1.7 (6)
O1—S1—C7—C8	−143.8 (2)	C12—O4—C11—C10	176.8 (4)
C5—S1—C7—C8	102.1 (3)	C9—C10—C11—O3	0.8 (6)
O2—S1—C5—C4	28.0 (3)	C9—C10—C11—O4	−177.7 (3)
O1—S1—C5—C4	159.4 (2)	C3—C2—C1—C6	0.0 (4)
C7—S1—C5—C4	−87.1 (3)	C3—C2—C1—Br1	−179.8 (2)
O2—S1—C5—C6	−155.5 (2)	C5—C6—C1—C2	−0.2 (4)
O1—S1—C5—C6	−24.1 (3)	C5—C6—C1—Br1	179.61 (19)
C7—S1—C5—C6	89.3 (2)	C1—C2—C3—C4	1.0 (5)
C4—C5—C6—C1	−0.5 (4)	C5—C4—C3—C2	−1.7 (5)
S1—C5—C6—C1	−176.9 (2)	C11—C10—C9—C8	−178.6 (3)
C6—C5—C4—C3	1.5 (5)	C7—C8—C9—C10	176.0 (3)
S1—C5—C4—C3	177.8 (2)	C11—O4—C12—C13	−156.5 (4)
S1—C7—C8—C9	−177.7 (2)		

### Hydrogen-bond geometry (Å, º)

**Table:**

*D*—H···*A*	*D*—H	H···*A*	*D*···*A*	*D*—H···*A*
C7—H7···O3^i^	0.93	2.37	3.235 (4)	154

Symmetry code: (i) −*x*+2, −*y*, −*z*.
